# Experiments with Mallein

**Published:** 1892-01

**Authors:** E. E. Bennett


### EXPERIMENTS WITH MALLEIN OR GLANDER LYMPH.*

By E. E. Bennett, F. R. C. V. S., A. V. D.

The paper by Dr. Pearson, which appeared in *The Veterinary Journal* for November,† is so interesting and important that I am led to enlarge upon the subject, and to give the most up to date details of the experiments that have recently been carried out in Germany, and the conclusions which have been arrived at by several investigators.

Koch's discovery of Tuberculin, and the specific reaction which it calls forth in tuberculous individuals, has opened up a new field of inquiry as to the treatment, diagnosis and prevention of infectious diseases generally; and however much the study may still be said to be in its infancy, yet there can be no doubt that in time, when the systems of culture and inoculation are perfected, this method will have a great practical importance in medicine. In the diagnosis of disease (the side of the question with which we are chiefly concerned at present), and more particularly in those diseases which, in their early and chronic stages, are sometimes most difficult of discernment, it will be of the utmost value in enabling a decision to be arrived at, and so doing away in a measure with the danger which may result from keeping animals for a length of time under observation.

Tuberculosis and glanders are maladies which may both be classed as difficult to diagnose in their early inception, and even in chronic cases the *intra vitam* appearances may be so little noticeable that we fail either to appreciate the gravity of the situation, or are unable to undertake the responsibility of a condemnatory decision.

Various methods for the diagnosis of glanders have from time to time been advocated, but they one and all are too tedious in operation, or too uncertain in result, to be of any real practical value ; some of these systems may be mentioned, *e. g.*, the inocula-

* *The Veterinary Journal*, December, 1891.

† The Journal of Comparative Medicine and Veterinary Archives, Vol. XII, No. 9, fol. 411, September, 1891.

tion of susceptible animals from such as are suspected; cohabitation; extirpation of glander and farcy nodules; destruction of farcy buds by the actual cautery; the artificial generation of fever to hasten on the diseased processes; the cultivation of the glanders bacillus, &c., &c. What we require in practice is a means which, being rapid, is also certain in its effects, and one which leads to no untoward results; following up, therefore, the discovery of tuberculin and the specific reaction which its inoculation calls forth in tuberculos subjects. Kalming, of Riga (since dead of glanders) and Hellman, of Dorpat, busied themselves in the production of a fluid which should produce a typical reaction in glandered animals. This material was manufactured (with slight deviations according to the dictates and experience of the experimenters) from potato cultures of the glander bacillus as follows: 5 grs. of the culture were thinned with 20 c.c.m. of sterilized water, and the mixture stood for twenty minutes in a Thermostat at 120°C., four times repeated during 24 hours, and the emulsion then obtained was further heated for 48 hours at a temperature of 39°C., and then with the help of an air pump passed through "Pasteur's filter." As a result, there was obtained about 12 c.c.m. of a light yellow fluid, which was yet again heated to 120°C. for 15 minutes, this oft-repeated heating being to insure the thorough destruction of the glanders bacillus. I must here point out that the glanders bacillus behaves in a *specific* manner when cultivated on the potato, changing the color of the culture ground from green to brown, and finally to black; no other known organism possesses a similar peculiarity when so prepared. The early experiments with mallein were carried out through the agency of guinea pigs, and the results obtained were very similar to those related by Dr. Pearson, viz., artificially glandered guinea pigs were injected with carbolised glandered lymph, and in each case such injection called forth a reaction, whereas in the control experiments on healthy guinea pigs, little or no rise in temperature was noted after inoculation. This success was followed up on the first opportunity by the inoculation of horses in a glandered stud, with a view of testing its efficacy as a means of diagnosis. The chance offered early in June last, where out of a stud of 11 farm horses, 5 had been destroyed as suffering from the disease, all of which on post-mortem examination showed manifest signs of glanders; the remaining 6 were, in consequence, ordered by the police authorities to be killed, but previous to the order being carried into effect, they were inoculated with mallein, the following being a short *resumé* of the experiment:

(1) Brown gelding, 4 years, poor condition, short cough, otherwise appearance healthy.

(2) Chestnut mare, 9 years, fair condition, short sharp cough.

(3) Chestnut mare, 8 years, low condition, soft cough.

(4) Chestnut mare, 15 years, weeping from left eye.

(5) Brown mare, 1¼ years, in good condition.

(6) Foal, 14 days, in good condition.

Nos. 1, 2, 3, 4, were injected with 0·3 c.c.m. of g. lymph.

No. 5, with 0·2 c.c.m. g. lymph

No. 6, with 0·1 c.c.m. g. lymph.

The inoculations were made under the skin of the throat or shoulder, with a sterilized syringe.

The following chart shows state of temperature and pulse after injection :

| June 10, 1891. | 1 | 2 | 3 | 4 | 5 | 6 |
|---|---|---|---|---|---|---|
| | 0·3 c.c.m. | 0·3 c.c.m. | 0·3 c.c.m | 0·3 c.c.m. | 0·2 c.c.m. | 0·1 c.c.m. |
| First injection— | Temp. P. | Temp. P. | Temp. P. | Temp. P. | Temp. P. | Temp. P. |
| 2 P.M. | 38·4°C. | 38·5°C. | 33·2°C. | 38·2°C. | 38·4°C. | 38·4°C. |
| 5 P.M. | 38·3,48 | 38·0,44 | 38·2,54 | 37·9,50 | 38·4,44 | 38·4 |
| 7 P.M. | 38·2,52 | 38·3,48 | 38·7,56 | 38·3,50 | 38·4,54 | 38·8 |
| Second injection— | 0·3 c.c.m. | 0·5 c.c.m. | 0·5 c.c.m. | 0·5 c.c.m. | 0·3 c.c.m. | 0·3 c.c.m, |
| 9 P.M. | 38·0,44 | 38·3,52 | 38·8,58 | 38·3,48 | 39·4,52 | 38·9 |
| 11th June, 5 A.M. | 40·1,60 | 39·8,54 | 40·4.64 | 39·4,56 | 49·1,48 | 38·6 |
| 7.30 A.M. | 40·4,60 | 39·6,56 | 39 0,60 | 39·7,58 | 39·1,54 | 38·4 |
| 9.30 A.M. | 39·8,60 | 39·6,48 | 39·6,60 | 39.5,56 | 39·3,48 | 38.0 |

Soon after the inoculation, a somewhat flat doughy swelling appeared at the point of injection in all the horses, with the exception of the foal (No. 6).

As the table shows, the temperature was highest in Nos. 1, 2, 3, 4 and 5, some 15 hours after the first, and some 8 hours after the second injection. In No. 1, the thermometer registered a total rise of 2°C., in No. 3, as much as 2·2°C., and in the remainder from 1·5°C. to 1·9°C. In the foal the temperature was only slightly elevated after the first, and, on the contrary, fell after the second injection. None of the animals refused their food, although the first five were manifestly drowsy, and disinclined to move after inoculation. On June 11, the orders for destruction were carried into effect, and a post-mortem examination was made in each case, and all with the exception of the foal (No. 6) were found to be glandered, typical lesions—both old and new—being brought to light.

As a control experiment, a mare condemned to be destroyed, as being useless for work, owing to the result of an accident, was inoculated with the same g. lymph as used in the other cases, and although as much as 1·8 c.c.m. was injected, no reaction occurred, nor was its general health in the least disturbed.

Since the foregoing, two other distinct sets of experiments have been undertaken by separate investigators, to test the efficacy claimed for the glander lymph as employed by Herr Preusse.

So lately as September last, Schilling, V. S., of Oppeln, carried out a series of inoculations in the remnant of a stud of 25 horses, 16 of which had been destroyed, and were proved on post-mortem to be suffering from glanders. The remaining 9, although carefully examined, appeared to be quite healthy; they were inoculated in a similar manner to that previously described, with 1·0 c.c.m. glander lympth, prepared by Herr Preusse himself and diluted before injection with one-tenth part of one per cent. carbolic solution; the first injection was made at 1 P.M., the second at 9.30 P.M. the same day.

TABLE SHOWING DETAILS OF EXPERIMENTS.

| No. | Description. | Before injection. T. | P. | R. | After first injection. T. | P. | R. | After second injection. Temp. | Result on P. M. examination. |
|---|---|---|---|---|---|---|---|---|---|
| 1 | Ch. M. 7 yrs. | 38·1 | 40 | 12 | 38·7 | 45 | 12 | 38·8 | Glandered. |
| 2 | Bn. G. 7 yrs. | 38·0 | 42 | 12 | 38 0 | 42 | 12 | 38·0 | Healthy. |
| 3 | Bn. G. 7 yrs. | 37·6 | 36 | 16 | 38·5 | 40 | 15 | — | Glandered. |
| 4 | Bn. M. 12 yrs. | 38·8 | 50 | 25 | 40·5 | 60 | 30 | 39·8 | " |
| 5 | Bn. G. 7 yrs. | 38·3 | 45 | 16 | 38·8 | 48 | 14 | 38·8 | " |
| 6 | Bn. G. 8 yrs. | 38·2 | 40 | 14 | 38·1 | 40 | 12 | 37·7 | Healthy. |
| 7 | Ch. G. 7 yrs. | 38·2 | 40 | 12 | 38·4 | 40 | 12 | 37·8 | " |
| 8 | Bn. M. 7 yrs. | 38·4 | 42 | 14 | 37·7 | 45 | 12 | 37·7 | " |
| 9 | Bn. M. 7 yrs. | 38·4 | 42 | 12 | 38.4 | 45 | 12 | 37·0 | " |

The first rise in temperature took place 8½ hours after the first inoculation, in four of the horses, viz., Nos. 1, 3, 4 and 5. In three of the horses, the rise was only some tenths of a degree centimetre, during the same time that No. 4 rose from 38·8°C. to 40·5°C.

In only one horse, No. 1, was there a further rise of temperature 8 hours after the second injection, and in no case was there acceleration of the pulse and respiration after the second injection.

The general appearance of the horses was not materially altered, except Nos. 3 and 4, which were for a short time off their feed and drowsy. The 9 horses experimented on were all shot at 8 A.M. the day following the injections, and examined by two independent Veterinary Surgeons, with the result that Nos. 1, 3, 4 and 5 were found to be glandered, whereas the others showed no suspicious signs of the disease whatever.

According to the table, it was proved that those horses which 8 hours after injection (except in No. 7, which varied only ·2°C.), had an increased temperature—*reaction*, were glandered, and that in two of these there was again an increase 8 hours after the second injection, and, further, that in the 5 horses in which little or no increase occurred after the first and second injections, there was not the least sign of glanders on P. M. examination.

In the case of the second series of experiments made by Peters and Felisch, I have unfortunately mislaid the details, but the general conclusions, which they were enabled to arrive at, were as follows, viz.:

(1) That Preusse's lymph, when injected into glandered horses, calls forth a *specific* reaction, which specially shows itself by an increase in the temperature, and that this reaction does not (* as a rule) take place in healthy animals.

(2) That the temperature elevation is not influenced by the severity of the already existing disease ; that is to say, the *reaction* is sometimes greater in those animals which appear to be suffering only slightly from glanders, than in those more seriously affected.

(3) The reaction sets in 8 to 10 hours after inoculation.

Granting that the outcome of the experiments hereinbefore mentioned is as accurate as we are led to suppose, by those who have undertaken the original researches in this direction, it is needless to remind the reader that the rapidity with which glanders could be identified, would be of inestimable service to the practitioner who has the responsible charge of large studs of animals, and who constantly has to be on the alert in interpreting any suspicious symptoms of this grave and incurable malady.

* In one case inoculated, a reaction took place, although the animal on P. M. was found free from glanders, but it may have been due to some constitutional disturbance, which passed unrecognized at time of injection.

